# Evolutionary history of black grouse major histocompatibility complex class IIB genes revealed through single locus sequence-based genotyping

**DOI:** 10.1186/1471-2156-14-29

**Published:** 2013-04-24

**Authors:** Tanja Strand, Biao Wang, Yvonne Meyer-Lucht, Jacob Höglund

**Affiliations:** 1 Population Biology and Conservation Biology, Department of Ecology and Genetics, Evolutionary Biology Center, Uppsala University, Norbyvägen 18D, Uppsala, SE-752 36, Sweden; 2 Current address: Swedish Institute for Communicable Disease Control, Department of Analysis and Prevention, Nobels väg 18, Solna, SE-171 82, Sweden

**Keywords:** Balancing selection, Concerted evolution, Early duplication, BLB1, BLB2, Galliformes, 3′UTR, *Tetrao tetrix*

## Abstract

**Background:**

Gene duplications are frequently observed in the Major Histocompatibility Complex (MHC) of many species, and as a consequence loci belonging to the same MHC class are often too similar to tell apart. In birds, single locus genotyping of MHC genes has proven difficult due to concerted evolution homogenizing sequences at different loci. But studies on evolutionary history, mode of selection and heterozygosity correlations on the MHC cannot be performed before it is possible to analyse duplicated genes separately. In this study we investigate the architecture and evolution of the MHC class IIB genes in black grouse. We developed a sequence-based genotyping method for separate amplification of the two black grouse MHC class IIB genes BLB1 and BLB2. Based on this approach we are able to study differences in structure and selection between the two genes in black grouse and relate these results to the chicken MHC structure and organization.

**Results:**

Sequences were obtained from 12 individuals and separated into alleles using the software PHASE. We compared nucleotide diversity measures and employed selection tests for BLB1 and BLB2 to explore their modes of selection. Both BLB1 and BLB2 are transcribed and display classic characteristics of balancing selection as predicted for expressed MHC class IIB genes. We found evidence for both intra- and interlocus recombination or gene conversion, as well as indication for positive but differential selection at both loci. Moreover, the two loci appear to be linked. Phylogenetic analyses revealed orthology of the black grouse MHC class IIB genes to the respective BLB loci in chicken.

**Conclusions:**

The results indicate that the duplication of the BLB gene occurred before the species divergence into black grouse, chicken and pheasant. Further, we conclude that BLB1 and BLB2 in black grouse are subjected to homogenizing concerted evolution due to interlocus genetic exchange after species divergence. The loci are in linkage disequilibrium, which is in line with the theory of tightly coevolving genes within the MHC under the minimal essential MHC hypothesis. Our results support the conclusion that MHC form and function in birds derived from studies on the domesticated chicken are not artefacts of the domestication process.

## Background

The duplication of genes is a common process in the evolution and adaptation of most organisms [1]. Understanding the molecular mechanisms that shaped duplicated genes is essential to study the evolutionary history and selection processes acting on complex gene families in a larger context. A very prominent example of a multigene family with frequent genetic and genomic duplications is the Major Histocompatibility Complex (MHC), which holds a key role in the vertebrate adaptive immune response in pathogen recognition and defense [2]. Thereby, MHC genes are linked to individual fitness and the outcome of infections (reviewed in e.g. [3]). The genes of the MHC are known to be the most polymorphic and among the best studied functional genes in vertebrates [4]. In humans, for instance, close to a thousand different alleles are known from the MHC HLA-DRB1 locus [5]. The extreme polymorphism commonly found at MHC genes is believed to be maintained by means of balancing selection, driven by selection through diverse pathogens and leading to maintenance of allelic variation within populations [6-8]. Balancing selection acts on variation at the MHC by means of heterozygote advantage (dominant as well as overdominant selection), rare-allele advantage (negative frequency-dependent selection) and/or selection that fluctuates over space and time [3]. Gene conversion and recombination [9], trans-species evolution [10], MHC-dependent sexual selection (reviewed in [4]) and selection against hitchhiking recessive deleterious mutations [11] have also been shown to play a role in shaping MHC diversity.

Duplications of MHC loci are frequently observed, and as a consequence loci belonging to the same MHC class are, in many species, too similar to distinguish by their allelic sequences [12-16]. However, studies on the evolutionary history, the mode of selection and heterozygosity correlations on the MHC cannot be performed before it is possible to analyse duplicated loci separately [4]. Single locus amplification of polymorphic MHC genes in birds has been a goal for evolutionary biologists for over a decade [12,17], but has not been achieved until recently. Very few bird species studied so far have only a single MHC class IIB locus (i.e. the green-rumped parrotlet [18], penguins [19] and kestrels [20]). Among the numerous birds with duplicated MHC class IIB loci, single locus amplification has so far only been reported in red jungle fowl/domestic chicken (*Gallus gallus*) [21,22], captive turkey (*Meleagris gallapavo*) [23] and Barn owl (*Tyto alba*) [24].

The MHC shows pronounced differences in genomic organization and in number of MHC loci between vertebrate lineages [25], especially when mammalian and non-mammalian species are compared [26,27]. Different hypotheses have emerged for how the complex MHC family has evolved. The prevailing consensus is that MHC evolution is characterized by repeated gene duplication (birth) and gene loss (death), whereby the loci evolve under a birth-and-death model [2,28,29]. Phylogenetic relationships can reveal whether gene duplication arose post speciation or pre speciation. Post speciation duplication leads to a pattern where paralogous MHC loci within a species are more closely related than orthologous MHC loci between species [30,31]. Pre speciation duplication, in contrast, took place in an ancestral species and results in orthologous MHC loci from different species being more closely related than paralogous MHC loci within species. However, these patterns can likewise be generated by different processes. A situation identical to what to be expected under a post speciation duplication event can be caused by concerted evolution acting on early duplicated genes [26,32]. Under concerted evolution, gene fragments are frequently exchanged between paralogous loci, and thereby homogenized (interlocus genetic exchange by recombination or gene conversion [9,33]). However, if convergent selection acts on either different loci within a species or between different species, those loci will functionally converge and mask the actual evolutionary relationship [32,34]. These different processes are not mutually exclusive, which adds a further level of difficulty to the interpretation of evolutionary patterns at the MHC.

The domestic chicken (*Gallus gallus domesticus*) is the major avian MHC model species with a history of more than 60 years of MHC studies [35]. The core of MHC in the chicken is called the classical MHC or the BF/BL region which, among others, consists of MHC class I and class II genes, TAP genes, Tapasin and one MHC class IV (BG) gene. This gives a total of 19 genes in 92 kb [36] situated on a single chromosome compared to the human MHC region which comprises 128 expressed genes as well as pseudo genes on a stretch of 3600 kb on several chromosomes [37]. The chicken MHC also appears more compact than what has been observed in the passerine bird which is best known, the zebra finch (*Taeniopygia guttata*), in which the MHC region is more complex and found on at least four chromosomes [38]. Because of the compact and small structure of the chicken MHC compared to the mammalian MHC, and the presence of few expressed genes, it has been named “a minimal essential MHC”. In addition, the chicken MHC is arranged differently than the mammalian MHC [36]. MHC class I and class II genes are tightly linked [31,39], and TAP genes has been demonstrated to co-evolve with MHC class I (BF) genes [31]. It has also been suggested that other MHC genes may co-evolve [30,40]. Based on experiments, recombination within BF/BL in the chicken is rarely observed [41] but sequencing showed evidence of gene conversion and recombination in shaping the chicken MHC [39]. Furthermore, examinations of BF/BL in pedigreed families in the closely related galliforms turkey and Japanese quail (*Coturnix japonica*) have revealed direct evidence of gene conversion and recombination [42,43].

In the chicken, both MHC class I (BF) and MHC class IIB (BLB) comprise two loci but only one locus in each class is highly expressed. While the BLB2 locus is considered to be dominantly expressed [44,45], the BLB1 locus has been suggested not be involved in peptide binding and to be neutral to selection [30,46]. Nevertheless, interlocus genetic exchange has been observed between BLB1 and BLB2 in a farmed population of pheasant (*Phasianus colchicus*) and in the domestic chicken, i.e. a case of concerted evolution [47]. In this study we test whether BLB1 and BLB2 evolved independently or in a concerted way in a wild close relative to both the pheasant and the chicken: the black grouse (*Tetrao tetrix*). Disentangling the evolutionary history of BLB1 and BLB2 in black grouse will add valuable information about how these genes evolved in the chicken and related species. To date, it is largely unknown if the uniquely compact MHC in the chicken [36] is a result of the long domestication process or was present before domestication.

**Figure 1:**
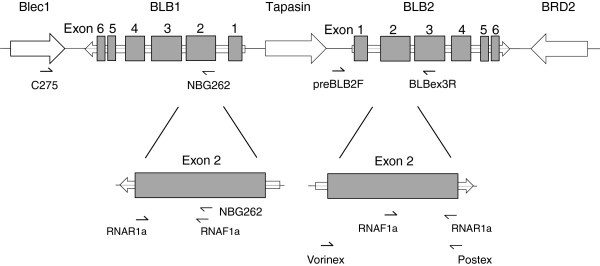
**Schematic figure of the genomic focus region of the MHC.** Genes are illustrated by white arrows indicating their orientation, with the names above the drawing. The exon-intron structure is given for BLB1 and BLB2 to show the target region and primer positions. The positions and amplification directions of all primers used in this study are indicated by small one-headed arrows. Below the upper part of the drawing, the primers for the long-range PCR amplifications of BLB1 and BLB2 (C275, NBG262 and preBLB2F, BLBex3R) are given. Further down, the target region Exon 2 is enlarged, with the positions of the nested PCR primers (BLB1: RNAR1a, RNAF1a, NBG262; BLB2: Vorinex, RNAF1a, RNAR1a, Postex) given below. Note that BLB1 and BLB2 are orientated in opposite directions.

Domestic chicken and black grouse belong to the same avian family (Phasianidae; Galliformes) and both possess two MHC class II B (BLB) loci that surround the Tapasin gene (Figure 1) [44,48]. Although the MHC class II B region is well studied in the domestic chicken, no primers could be developed for independent amplification of either MHC class II B minor (BLB1) or MHC class II B major (BLB2) [49]. The chicken BLB1 and BLB2 are nearly identical in sequence and can only be amplified separately with PCR primers anchored outside the BLB genes along with a nested non-locus-specific PCR [22,44].

In this study, we present an approach to genotype the two MHC class II B loci separately in a wild galliform, the black grouse. The procedure comprises a long-range anchored PCR method followed by nested PCR reactions and sequence-based genotyping. Based on this approach we first aim to characterize and contrast MHC class II B diversity and mode of selection at the BLB1 and BLB2 loci in the black grouse. Second, we explore whether the two loci underlie concerted evolution or evolve independently. And third, we relate and compare the results to the MHC class II B structure and organization in the chicken.

## Methods

### Samples

We studied locus-specific MHC class IIB variation in twelve black grouse individuals, eleven of which were genotyped non locus-specific in our previous studies [15,50]. We included one more individual (JHGO 213) and genomic DNA was extracted from tissue with a salt extraction protocol [51]. In addition, we used sequence data obtained in a fosmid sequencing study on the same individual JHGO 213 [48] [GenBank JQ028669]. Sequences from other species used in the data analysis derived from GenBank, accession numbers are given in the respective figure legends.

### Locus-specific amplification of BLB1 and BLB2

In order to amplify the two black grouse MHC class IIB genes BLB1 and BLB2 separately, we applied a long-range anchored PCR method combined with nested non locus-specific PCRs. For each BLB locus, we anchored one of the PCR primers outside of the respective BLB gene for a locus-specific long-range PCR (Figure 1). In a second step, these long-range PCR fragments were used as template in nested PCRs with non locus-specific primers to amplify parts of the BLB exon 2.

We selectively amplified a long fragment of the BLB1 gene with a modification of primers designed for the chicken *BLB minor*[44]. We used the forward primer C275 located in the Blec1 gene adjacent to BLB1 gene, and the reverse primer NBG262, slightly modified from C262 situated within BLB1 exon 2 (Figure 1) (see Additional file 1 for primer sequences). These primers yielded a 1932 bp PCR product. All attempts to design primers amplifying the complete BLB1 exon 2 based on chicken genome sequences or on black grouse MHC fosmid data failed. In the 20 μl BLB1 long-range reaction, approximately 100 ng DNA, 0.3 μM of each primer C275 and NBG262, 0.8 μM dNTP, 0.6 μl DMSO, 1x Phusion GC buffer and 1U Phusion High-Fidelity DNA polymerase (Finnzymes, Espoo, Finland) were used. The PCR programme was initiated with 40 s at 98°C, followed by 35 cycles of 10 s at 98°C, 20 s at 62°C and 68 s at 72°C and ended with 6 min at 72°C.

Based on the black grouse MHC fosmid data [GenBank JQ028669] [48] we designed primers for a selective amplification of the BLB2 gene. We imported the BLB2 sequence and flanking regions to the NCBI web based primer design program Primer BLAST [52] and marked areas that were conserved between the black grouse BLB2, the chicken *BLB major* and the turkey BLB2 (*Meleagris gallopavo*), but were different from BLB1. The resulting primer pair forward preBLB2F and reverse BLBex3R yielded a 1359 bp PCR product ranging from the intragenic region between the Tapasin gene and BLB2 to the BLB2 exon 3 (Figure 1) (see Additional file 1 for primer sequences). For amplification of the BLB2 we used approximately 100 ng DNA, 0.3 μM of each primer preBLB2F and BLBex3R, 0.3 μM KAPA dNTP, 1x KAPA GC buffer and 0.5 U KAPA HiFi DNA polymerase (KAPA BIOSYSTEMS, Boston, United States) in a 25 μl reaction. The PCR programme was initiated with 5 min at 98°C, followed by 35 cycles of 20 s at 98°C, 15 s at 62°C and 90 s at 72°C and ended with a final extension for 5 min at 72°C.

Long-range PCR products for BLB1 and BLB2 revealed each one strong single band on a 1.5% agarose gel. A sterile toothpick was inserted into each band, taking up a small amount of the PCR product, and transferred into 50 μl ddH_2_0. This solution was stored overnight at -20°C before using it in the nested PCRs as template as described in the following.

### Nested PCR to amplify exon 2 and sequencing

The primers RNAF1a and RNAR1a [15] (Additional file 1) were used in a nested PCR to amplify 125 bp on the BLB1 and BLB2 long-range PCR products as template. 1 μl of the template solution, 0.48 μM of each primer, 0.6 mM of dNTP, 3 mM MgCl_2_, 1 x buffer and 0.75 U BioTaq DNA polymerase (DNA Technology, Aarhus, Denmark) were used in a 25 μl PCR reaction. The PCR programme was initiated at 94°C for 5 min, followed by 30 cycles at 94°C for 1 min and 30 s at both 64.9°C and 72°C, and ended for 10 min at 72°C. We also performed another nested PCR amplifying a longer part of the exon 2 on the BLB2 long-range PCR product only. A second primer pair, Vorinex2 and Postex2, developed for the closely related willow grouse (*Lagopus lagopus*), was used to amplify 251 bp of exon 2 (see Additional file 1). 1 μl of the template solution, 0.4 μM of each primer, 0.48 mM of dNTP, 1 x KCl buffer and 1 U Taq DNA polymerase (Fermentas, St. Leon-Rot, Germany) were used in a 25 μl PCR reaction. The programme was initiated at 94°C for 5 min, followed by 30 cycles at 94°C for 1 min and 30 s at both 66°C and 72°C before a final extension for 10 min at 72°C. Using the primer pair Vorinex2/Postex2 a longer portion of BLB2 exon 2 sequences were amplified that were not known from before, so every new 251 bp sequence was subjected to verifying PCR runs (both BLB2 long-range PCR and nested PCR) and sequencing. For BLB1, the 251 bp nested fragment could not be amplified due to the position of the nested primer Vorinex2 outside of the BLB1 long-range PCR product.

The nested PCR products were cleaned with an Exo-Sap reaction (Exonuclease I-Shrimp Alkaline Phospate, Fermentas, St. Leon-Rot, Germany). Cycle sequencing reactions were performed in both directions and the sequencing products were subjected to a post-reaction clean-up (GE Healthcare, Uppsala, Sweden), following the protocols of the manufacturer. All direct sequencing was performed both forward and reverse on a MegaBACE 1000 DNA analysing system (GE Healthcare, Uppsala, Sweden).

### Confirmation of MHC alleles

PCR artefacts are a large issue in MHC studies and cautions to reduce the formation of artificial alleles are important to address [53]. To decrease the probability of PCR artefacts we only regarded alleles present in two independent PCR reactions as confirmed [54]. For the long-range PCR reactions for BLB1 and BLB2 we used different enzymes (Phusion High-Fidelity DNA polymerase and KAPA HiFi DNA), which could produce different levels of PCR artefacts. We were able to compare the sequences derived by the long-range PCR presented here, with previously cloned sequences using the non locus-specific primers RNAF1a and RNAR1a directly on genomic DNA [15,50]. Between 16 and 26 clones were sequenced per individual in the previous studies. We found that enzymes in the long-range PCRs of BLB1 and BLB2 worked equivalently well in amplifying alleles corresponding to those previously found (Table 1).

**Table 1:** Allele designations for the twelve black grouse individuals included in this study

**Individual**	**BLB1**_**125**_	**BLB2**_**125**_	**BLB2**_**251**_	**Summary**	**BLB cloning**
				**BLB1 & BLB2**	Non-specific*
D248	06	07, 12	07, 12	06, 07, 12	04, 06, 07, 12
D249	06, 11	09, 10	09, 10	06, 09, 10, 11	06, 11
D320	03, 06	01, 07	01A, 07	01A, 03, 06, 07	01, 06, 07
D375	05	01	01A, 01B	01A, 01B, 05	01, 05
D476	05	01, 09	01A, 09	01A, 05, 09	01, 05, 09
D870	03, 04	01, 02	01A, 02	01A, 02, 03, 04	(cDNA) 01, 02, 03, 04
D115804	04, 14	01, 04	01C, 04B	01C, 04, 04B, 14	01, 04
H070	05, 06	01, 09	01B, 09	01B, 05, 06, 09	01, 05, 06
H071	04, 05	01, 02	01B, 02	01B, 02, 04, 05	01, 02, 04, 05
H369	05	01, 02	01A, 02	01A, 02, 05	01, 02, 05
H393	05, 06	01, 04	01B, 04B	01B, 04B, 05, 06	01, 04, 05, 06
JHGO213	04	01, 02	01A, 02	01A, 02, 04	no
**H**_**obs.**_	0.58	0.92	1		
**N of alleles**	6	7	9	minimum 14	11

*Previous non locus-specific cloning from [15,50].

### Data analysis

#### Identification of sequences and analysis of sequence variation

CodonCode Aligner version 3.7.1 was used for sequence editing and performing ambiguity codes for heterozygous positions. Allelic phase for heterozygote sequences was determined by computational inference with the PHASE haplotype reconstruction algorithm in DnaSP version 5.10.05 [55]. The PHASE algorithm has been proven reliable for determining the allelic phase in a number of previous MHC studies (e.g. [56,57]). The parameters used were 1000 iterations, thinning 10, 100 burn in, and a recombination model. For the haplotype reconstruction of the 125 bp fragment we included sequences that were previously verified by cloning. Sequence variation statistics were calculated in DnaSP version 5.10.05 [55] including the number of segregating sites, nucleotide diversity (π) and average number of nucleotide differences (theta *k*).

#### Recombination, gene conversion and linkage

The power of any method for the detection of recombination or gene conversion strongly depends on the number of sequences included, the recombination rate and the number of sites differentiating the recombinant sequences [58,59]. Therefore it is suggested to apply an array of different methods to analyse recombination. We used a set of seven methods to detect recombination signals and putative recombinant sequences. The minimum number of recombination events (R_m_) according to the four-gamete test by Hudson & Kaplan [60] was calculated in DnaSP version 5.10.05 [55]. This method tests for overall evidence of recombination in the alignment. We used the RDP3 package [61] to apply the methods RDP [62], Maxchi [63], Chimaera [64] and GENECONV [65]. These methods are designed to detect recombination breakpoint locations; the first three use a dynamic sliding window, whereas GENECONV searches for unusual long regions of identity between sequences. Moreover, we used the method GARD [66] implemented in Datamonkey [67], which likewise identifies recombination breakpoints, searching all possible partitions in a probabilistic way. Recombination rate ρ and mutation rate θ were calculated using LDhat recombination rate scan [68].

We tested for linkage between the BLB1 and the BLB2 locus applying a likelihood ratio test of linkage disequilibrium implemented in Arlequin 3.5 [69]. In this test, the likelihood of the sample evaluated under the hypothesis of no association between loci (linkage equilibrium) is compared to the likelihood of the sample when association is allowed (linkage disequilibrium).

### Analysis of selection

Positive selection is the spread to fixation of an allele that increases the fitness of individuals [70]. Initially, a locus undergoing balancing selection seems to be subjected to positive selection, but then experiences negative selection on alleles that became too frequent (under frequency dependent selection). Testing for long-term balancing selection in genetic regions therefore often includes testing for positive selection, as inferred from the ratio of non-synonymous/synonymous (*dN/dS* or ω) substitution rates, for instance [71]. Therefore, the outcome of methods can reflect a positive selection process, although in our context, testing for positive selection within a species is translated into balancing selection.

Tajima’s D, Fu and Li’s D and F tests were analysed in DnaSP version 5.10.05 [55] and Fisher exact tests were employed to test for differences from neutrality. In addition to the averaged Tajima’s D, we performed sliding window Tajima’s D with a window size of 11 bp and a step size of 1 bp for the 125 bp fragments of the black grouse BLB1 and BLB2. A negative Tajima’s D (as Fu and Li’s D, Fu and Li’s F) is a sign of negative selection or population expansion and a positive value is a sign of positive/balancing selection or a population bottleneck. Averaged synonymous (*dS*) and non-synonymous (*dN*) substitutions per synonymous and non-synonymous site were calculated in MEGA 5.05 [72]. The Nei-Gojobori method with Jukes Cantor corrections and 5000 bootstrap replicates was used to calculate the overall average of *dN/dS* (ω) for all sites, the peptide binding region (PBR) and non-PBR according to [73]. Deviations from neutrality (*dN* = *dS*) were assessed by a Z-test. Differences in *dN* and *dS* between PBR and non-PBR were tested for significance using Mann–Whitney *U*-tests.

A different approach to detect molecular evidence of positive selection is to calculate ω per codon using the maximum likelihood method CODEML implemented in PAML version 4.6 [74]. The programme estimates heterogeneous ω among sites applying different models of codon evolution. We compared M2a (allowing ω to vary between ω <1 (conserved), ω = 0 (neutral) and ω > 1 (positive)) with M1a (ω allowed to vary between ω <1 and ω = 0) and M8 (ω can vary in beta distribution of 0 and 1 including ω > 1) with M7 (ω allowed to vary in a beta distribution of 0 and 1) using a log-likelihood test. Significant positive selection was inferred if twice the difference in log-likelihood values between the two models was greater than the χ^2^ critical value for the given degrees of freedom. The Bayes empirical Bayes (BEB) approach was used to identify significantly positively selected codon sites. Tree files used in PAML analyses were generated using a maximum likelihood approach in PhyML3.0 [75], under the F81 model of nucleotide substitution and estimated gamma shape parameter. Models of nucleotide substitution and the distribution of rate variation across nucleotide sites (gamma) were estimated in jModelTest 0.1.1 [76]. The best molecular evolution models were selected by the Aikaike information criterion (AIC) (see Additional file 2).

### Reconstruction of phylogenetic relationships

We constructed phylogenetic networks based on the BLB exon 2 sequences and on the 3^rd^ codon positions of exon 2 only to illustrate the phylogenetic relationship among the black grouse BLB1 and BLB2 alleles and possible orthology to the chicken BLB1 and BLB2. The networks were built with the software SplitsTree4, using the neighbour net method [77]. Phylogenetic trees based on exon 3 sequences and on 3′ untranslated regions (UTRs) were constructed using the Neighbour-joining method with bootstrapping (1000 replicates) implemented in MEGA 5.0 [72]. For BLB orthology between the black grouse and the chicken, we particularly looked at the 3′UTRs, since orthology between the pheasant and the chicken BLB1 and BLB2 has previously been demonstrated with the 3′UTRs [47].

## Results

### Locus-specific genotyping of black grouse BLB

**Figure 2:**
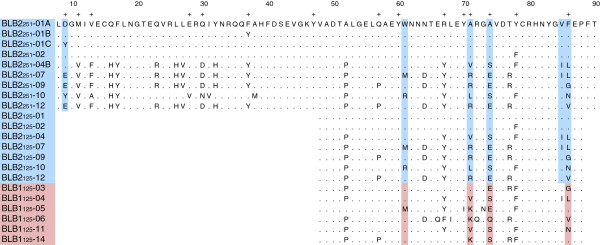
**Alignment of amino acid sequences.** PBR positions from Tong et al. [73] are marked with a +. The shaded amino acids are the PAML derived positively selected codon positions, blue for BLB2 and pink for BLB1.

We successfully amplified locus-specific long-range PCR products for both BLB1 and BLB2 in all twelve black grouse individuals. In subsequent nested PCR reactions, we amplified and sequenced a 125 bp PBR-rich fragment of the exon 2 for both loci, and almost the whole exon 2 for the BLB2 locus (251 out of 270 bp). Positions and sequences of the different primers are given in Figure 1 and Additional file 1. All heterozygous sequences could be assigned to haplotypes using the software PHASE implemented in DnaSP [55]. At the BLB1 locus we detected 6 unique 125 bp alleles (BLB1_125_, Table 1, Figure 2) and at the BLB2 locus 7 unique 125 bp alleles (BLB2_125_) and 9 unique 251 bp alleles (BLB2_251_). None of these sequences contained an indel or a stop codon, we thus assume that they are functional.

At the BLB1_125_ locus seven out of twelve individuals were heterozygous, whereas at the BLB2 all but one individual (BLB2_125_), respective all individuals (BLB2_251_) were heterozygous (Table 1). For allele BLB2_125_-01 the 251 bp fragment revealed additional variation towards the 5′-end of exon 2. This resulted in a subdivision into BLB2_251_-01A, BLB2_251_-01B and BLB2_251_-01C, each differing by one amino acid exchange (Figure 2). We found one 125 bp-allele that occurred at both BLB loci, namely BLB_125_-04. There is indication, however, that BLB_125_-04 differs at the 5′-end of exon 2 between the two loci [see Additional file 3, BLB2_251_-04B and BLB_202_-04-cDNA, the latter from our previous study [15].

All individuals displayed unique MHC class II B genotypes. Combining the data for BLB1 and BLB2, 12 individuals had a minimum of 14 unique BLB alleles and each individual carried between three and four alleles. In comparison, 11 out of the 12 individuals previously analysed by non locus-specific BLB cloning [15,50] revealed 11 unique BLB alleles and individuals carried between two and four alleles (Table 1). This was a minor underestimation of both the number of unique alleles and the number of alleles per individual due to the applied method compared to the here presented more accurate single locus amplifications.

There is evidence that both BLB loci are expressed in the black grouse. In an earlier study we found four BLB sequences in one individual by non locus-specific cloning based on cDNA (D870, Table 1, [15]). Our current study reveals that two of these sequences stem from BLB1 and the other two from BLB2.

### Sequence analyses: gene diversity, recombination and linkage

Although the locus BLB1_125_ showed a lower number of unique alleles than BLB2_125_, it revealed more segregating sites, a higher number of average pairwise differences theta *k* and a higher nucleotide diversity π (Table 2). As expected, the longer fragment BLB2_251_ revealed more alleles, more segregating sites, a higher theta *k* but a lower π than the shorter BLB2_125_. For BLB1_125_, 7 out of 8 positions involved in antigen binding [73] were polymorphic, whereas 6 out of 8 and 12 out of 15 positions were variable for BLB2_125_ and BLB2_251_, respectively (Figure 2).

**Table 2:** Gene diversity measures

**Locus**	**A**	**S**	**Theta *k***	**π**	**Tajima’s D**	**Fu & Li’s D**	**Fu & Li’s F**
BLB1_125_	6	34	15.87	0.13	-0.74	-0.52	-0.62
BLB2_125_	7	23	12.29	0.09	0.43	0.91	0.88
BLB2_251_	9	43	20.42	0.08	0.56	0.53	0.60
BLB1&2_125_	12	35	13.79	0.11	-0.25	-0.01	-0.08

Number of unique alleles (A), number of segregating sites (S), average pairwise differences (theta *k*), nucleotide diversity (π) and neutrality test summaries for the different fragments of BLB1 and BLB2. All neutrality tests were non-significant (all p > 0.10).

We used a set of different methods to detect recombination signals and putative recombinant sequences, and found evidence for recombination in each of the fragments (BLB1_125_, BLB2_125_, BLB2_251_, BLB1&2_125_), at least with the four-gamete test and the method Maxchi (Table 3). These recombination signals lead to a number of recombinant sequences including both intra- and interlocus recombination or gene conversion (Table 4). Considering both loci together (BLB1&2_125_) the estimated population recombination rate ρ was 11.42, and the mutation rate θ was 9.27, leading to a recombination-mutation ratio of 1.23. A ρ/θ ratio of > 1 is an indication for recombination being more prevalent in the dataset than point mutations [78]. For the loci separately, however, the estimated mutation rates were much higher than ρ, leading to ρ/θ ratios < 1. The results of the different tests combined strongly suggest that recombination or gene conversion has occurred in the black grouse MHC class II.

**Table 3:** Number of recombination events calculated by different methods

**Locus**	**No. of sequences**	**R**_**M**_	**RDP**	**GENECONV**	**Maxchi**	**Chimera**	**GARD**	**ρ**	**θ**	**ρ/θ**
BLB1_125_	6	4	0	0	2	1	2	5.45	12.26	0.44
BLB2_125_	7	3	0	0	1	0	1	4.02	7.76	0.52
BLB2_251_	9	6	0	1	1	1	0	1.37	13.61	0.10
BLB1&2_125_	12	6	0	0	2	1	1	11.42	9.27	1.23

R_M_, RDP, GENECONV, Maxchi, Chimaera and number of recombination breakpoints calculated GARD. Recombination rate (ρ), mutation rate (θ) and the ratio (ρ/θ) were calculated using LDhat.

**Table 4:** Recombinant sequences and their potential parental sequences at the black grouse BLB1 and BLB2 (calculated with Maxchi)

**Locus**	**Recombinant sequence**	**Major parent**	**Minor parent**
BLB1_125_	BLB1_125_-03	BLB1_125_-06	BLB1_125_-14
	BLB1_125_-04, BLB1_125_-11, BLB1_125_-14	BLB1_125_-06	BLB1_125_-05
BLB2_125_	BLB2_125_-07, BLB2_125_-09, BLB2_125_-12	BLB2_125_-04	unknown
BLB2_251_	BLB2_251_-04B	BLB2_251_-07	BLB2_251_-10
BLB1&2_125_	BLB1_125_-05	BLB2_125_-07	BLB2_125_-02
	BLB2_125_-07, BLB2_125_-09, BLB2_125_-12, BLB1_125_-03	BLB1_125_-06	BLB2_125_-01

We observed significant linkage disequilibrium between the BLB1 and BLB2 (likelihood-ratio-test, LnLikelihood LD: -46.087, LnLikelihood LE: -62.120, exact p = 0.0239), indicating that the two loci are linked.

### Analyses of selection

**Figure 3:**
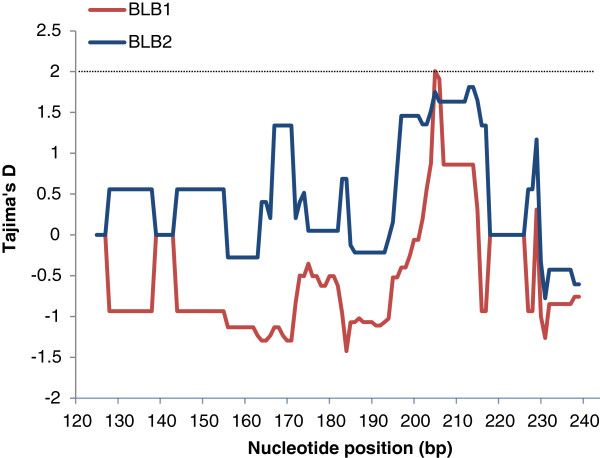
**Sliding window Tajimas’ D, for the 125 bp exon 2 fragments of the black grouse BLB1 and BLB2 (window size 11 bp, step size 1 bp).** The threshold for P < 0.05 is shown by the dotted line.

Positive values for Tajima’s D, Fu & Li’s D and Fu & Li’s F were detected for BLB2_125_ and BLB2_251_ (Table 2), which are an indication for positive or balancing selection acting on the BLB2 locus. On the other hand, we found these values negative for BLB1_125_ and the combination of BLB1&2_125_, which is generally a sign of negative or purifying selection. None of these values were significant, however, so that they have to be interpreted as indication rather than evidence. A sliding window analysis of Tajima’s D along the BLB1_125_ and BLB2_125_ further disentangled this pattern of differences in selection at specific sites between the two loci (Figure 3).

We calculated the relative rates of non-synonymous (*d*_*N*_) and synonymous (*d*_*S*_) substitutions for all sites, the PBR and the non-PBR according to Tong et al. (2006) (Table 5). For the PBR, the values for the ratio *d*_*N*_/*d*_*S*_ were considerably larger than 1 in all fragments, which was significant in the case of BLB2_251_. This is considered to be evidence for positive selection acting on the PBR. Moreover, both *d*_*N*_ and *d*_*S*_ were significantly higher at the PBR sites compared to the non-PBR sites for all fragments (Mann–Whitney *U*-test, all p < 0.01), a pattern which has recently been explained to be created by gene conversion combined with positive selection [78]. Synonymous mutations hitchhike alongside beneficial non-synonymous mutations and thereby occur more frequent than under neutral expectations.

**Table 5:** **Relative rates of non-synonymous (*d***_***N***_**) and synonymous (*d***_***S***_**) substitutions with standard errors calculated for the two MHC class IIB loci, averaged over all sites, the peptide binding region (PBR) and non-PBR according to Tong et al.**[73]

**Positions**	**Locus**	***d***_***N***_ **±** ***SE***	***d***_***S***_ **±** ***SE***	***d***_***N***_***/d***_***S***_	**Z**	**p**
**All**	BLB1_125_	0.14 ± 0.04	0.17 ± 0.05	0.80	-0.61	0.54
	BLB2_125_	0.11 ± 0.04	0.11 ± 0.05	1.00	0.01	0.99
	BLB2_251_	0.11 ± 0.04	0.07 ± 0.02	1.46	1.06	0.29
	BLB1&2_125_	0.12 ± 0.04	0.14 ± 0.05	0.88	-0.33	0.74
**PBR**	BLB1_125_	0.57 ± 0.22	0.31 ± 0.21	1.84	1.37	0.17
	BLB2_125_	0.53 ± 0.24	0.27 ± 0.20	1.95	1.05	0.30
	**BLB2**_**251**_	**0.41 ± 0.12**	**0.16 ± 0.10**	**2.55**	**2.16**	0.03*
	BLB1&2_125_	0.54 ± 0.20	0.29 ± 0.20	1.84	1.23	0.22
**non PBR**	BLB1_125_	0.06 ± 0.02	0.14 ± 0.06	0.42	-1.64	0.10
	BLB2_125_	0.04 ± 0.02	0.08 ± 0.04	0.56	-0.85	0.41
	BLB2_251_	0.05 ± 0.01	0.05 ± 0.02	0.92	-0.15	0.88
	BLB1&2_125_	0.05 ± 0.02	0.11 ± 0.04	0.47	-1.58	0.18

Positive selection on specific codon sites was detected using the maximum likelihood method CODEML implemented in PAML4.6 [74]. Two pairs of models were applied: M2a versus M1a, and M8 versus M7. The models M2a and M8, which allow for positive selection, fitted the data significantly better than the neutral models for both BLB loci (Table 6 and Additional file 4). Several codons were identified as significantly positively selected (ω > 1, Table 6 and Figure 2). Four selected codons were identified with both M2a and M8 for BLB1_125_ and four and five codons were identified with M2a and M8, respectively, for BLB2_125_. Codons 71, 74 and 86 were identified as positively selected in both BLB1_125_ and BLB2_125_. In addition, position 67 was identified in BLB1_125_ and position 61 and 85 in BLB2_125_. The longer fragment BLB2_251_ confirmed the same positively selected codons as calculated for the fragment BLB2_125_, and revealed one additional position at the 5′-end of the molecule (position 9). All identified positively selected codon positions were identical or directly situated next to a peptide binding site as identified by Tong et al. [73].

**Table 6:** Summary table for CODEML

**Locus**	**Model (ref)**	**2(L**_**b**_**-L**_**a**_**)**	***p***	**Positively selected codons**					
				**9**	**61**	**71**	**74**	**85**	**86**
BLB1_125_	M1a - **M2a**	34.48	<0.001		**x**	**x**	**x**		**x**
	M7 - **M8**	35.56	<0.001		**x**	**x**	**x**		**x**
BLB2_125_	M1a - **M2a**	22.90	<0.001		**x**	**x**	**x**		**x**
	M7 - **M8**	23.02	<0.001		**x**	**x**	**x**	**x**	**x**
BLB2_251_	M1a - **M2a**	27.08	<0.001		**x**	**x**	**x**		**x**
	M7 - **M8**	27.12	<0.001	**x**	**x**	**x**	**x**	**x**	**x**
BLB1&2_125_	M1a - **M2a**	45.40	<0.001		**x**	**x**	**x**	**x**	**x**
	M7 - **M8**	48.38	<0.001		**x**	**x**	**x**	**x**	**x**
	Dist. to Tong PBR			0	1	0	0	1	0

Two model comparisons were conducted; each comparison includes a neutral model (M1a, M7) and a model allowing for positive selection (M2a, M8). Significance was assessed by comparing twice the difference in likelihood 2(L_b_-L_a_) between the models to a *χ*^2^-distribution (df = 2). The models fitting our data best are marked in bold. x marks positively selected codons calculated by Bayes Empirical Bayes (BEB) at the >95% confidence level. Distance (Dist.) to Tong PBR corresponds to the amino acid distance between identified site and the nearest peptide binding position identified by Tong et al. [73]. For parameter estimates see Additional file 4.

### Reconstruction of the phylogenetic relationships

**Figure 4:**
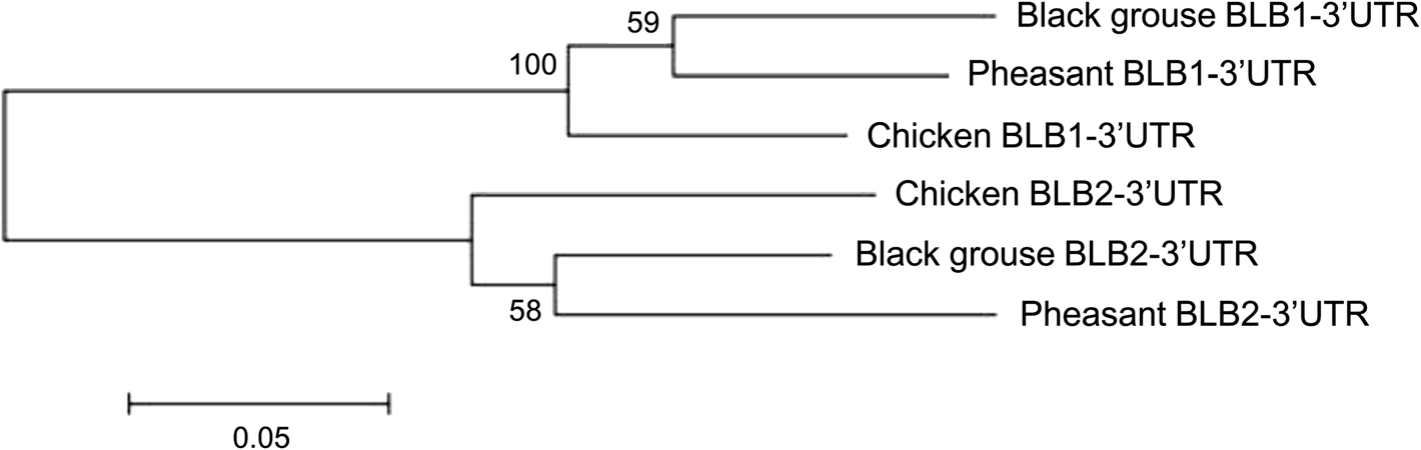
**Neighbour joining tree for the 3′UTR.** BLB1 and BLB2 sequences derived from black grouse (fosmid individual JHGO 213 [48], chicken [GenBank AB268588] and pheasant [GenBank AJ224349]*.* The scale bar represents substitutions per site.

In order to take a closer look at the orthology of the black grouse BLB1 and BLB2 with the corresponding BLB loci in the chicken and pheasant, we constructed a phylogenetic tree of the BLB1 and BLB2 3′UT regions (Figure 4), which are untranslated DNA sequences containing regulatory sequences. It has been shown that the BLB loci in both chicken and pheasant can be clearly distinguished by this part of the gene [47], in terms of nucleotide divergence and length. We found that the 3′UTR’s of the three species cluster according to gene and not according to species, forming one distinct BLB1 cluster and one BLB2 cluster, and similar differences in length. This suggests orthology of the BLB1 gene between the black grouse, chicken and pheasant, and orthology of the BLB2 gene between the three species.

**Figure 5:**
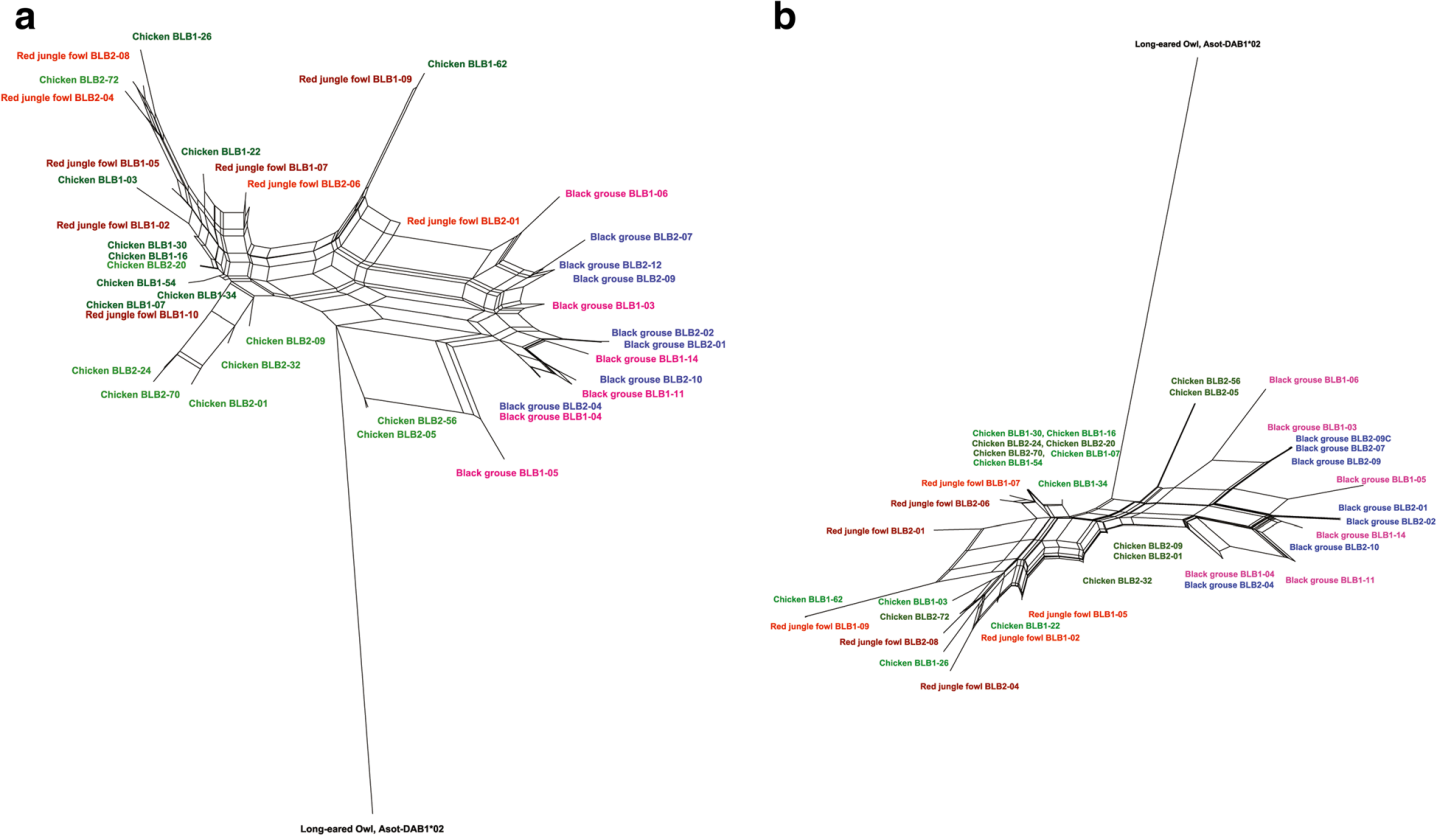
**Neighbour network for BLB1 and BLB2.** Sequences of the black grouse (pink and blue), chicken (dark and light green) [GenBank AB426144, AB426150-51, AJ248577, M29763] and red jungle fowl (brown and orange) [GenBank AM489767 - AM489776] for the **a**) 125 nucleotide sequence of exon 2 and **b**) only 3^rd^ codon positions in the 125 nucleotide sequence of exon 2. Long-eared Owl Asot-DAB1*02 [GenBank EF641225] was used as an outgroup.

A contrasting phylogenetic relationship is shown in the neighbour network of the exon 2 sequences of BLB1 and BLB2 (Figure 5a). Here, sequences cluster according to species and not according to gene. All black grouse sequences cluster together, with BLB1 and BLB2 sequences intermingling. The black grouse cluster is distinct from the sequences of the domestic chicken and red jungle fowl, which form a mixed cluster, again BLB1 and BLB2 intermingling. The same picture is apparent in a neighbour network based on the third codon positions of exon 2, at which variation is mostly synonymous and therefore likely neutral. Further, exon 3 sequences of BLB1 and BLB2 repeat the same clustering according to species (see Additional file 5).

## Discussion

The chicken MHC stands out with an unparalleled simple and compact architecture compared to mammals or other avian groups like the passerines [26,36]. The tight linkage of MHC genes and reduced recombination rates were suggested to have resulted in a close co-evolution of genes within MHC haplotypes ([40] but see [39]), leading to strong disease-associations of specific MHC haplotypes in consequence. In the evolutionary history of the avian MHC it is still not resolved whether this unique MHC structure in the chicken is a domestication artefact or was present before domestication as a galliform feature.

We here present a study on diversity, selection patterns and the co-evolutionary history of two transcribed MHC class IIB genes in a wild galliform bird species, the black grouse. We found evidence for both intra- and interlocus recombination or gene conversion, as well as indication for positive selection on the PBR at both loci. However, we also detected differences in the selection between the two loci, as positive selection was indicated at the BLB2 locus and purifying selection at the BLB1 locus. We were able to relate our findings to the structure of the chicken MHC class II by resolving orthology to the chicken BLB loci.

Similar to the work of [22] on red jungle fowl, we amplified the two black grouse BLB loci separately in twelve individuals. Eleven of the individuals included in this study were cloned in our previous studies with non locus-specific primers that gave a 125 bp BLB exon 2 product [15,50]. The resulting sequences showed that individuals carried between two and four BLB alleles, without locus assignment. This is a slight underestimation in number of alleles per individual compared to the here presented study, which revealed between three and four alleles per individual for BLB1 and BLB2 combined. Part of this additional variation was detected through the longer BLB2 fragment, which revealed further variable positions towards the 5′-end of the exon 2. To another part, the underestimation by the cloning/sequencing approach may be a result of too few analysed clones (16–26 per individual). On the other hand, for individual D248 the cloning procedure revealed one allele (BLB*_125_-04) which could not be confirmed by the single-locus amplification. This additional allele is likely to be a contamination during the cloning process. Considering that cloning is more time-consuming than direct sequencing, can add artefacts derived from mismatch repair or the formation of chimeric sequences [53] and is more contamination-prone, the here presented single locus sequence-based typing method clearly outmatches the cloning/sequencing approach in both accuracy and effort.

Another finding in this study concerns the expression of the two BLB loci in black grouse. We earlier amplified BLB cDNA sequences for individual D870 [15], which we could show now derived from both BLB1 and BLB2. Thus, both BLB1 and BLB2 are transcribed loci in black grouse. In domestic chicken BLB2 is considered to be dominantly expressed [44,45], while BLB1 is less expressed and has even been suggested to be neutral to selection and not involved in peptide binding [30,46]. This ultimately leads to the question whether the BLB1 and BLB2 loci in black grouse show a similar situation or differ in the degree and mode of selection compared to the chicken. We therefore compared diversity and selection patterns between BLB1 and BLB2 to explore their respective modes of selection.

We observed significant linkage between BLB1 and BLB2, in line with the hypothesis of Kaufman [30,40] that MHC genes in chicken form a tightly linked cluster of co-evolving genes. Similarly, Agudo et al. [79] showed in a study on Egyptian vultures (*Neophron percnopterus*) the existence of linkage groups, containing pairs of MHC alleles in strong linkage disequilibrium. They interpret the presence of linkage groups with similar MHC alleles as indication of concerted evolution acting on the MHC gene duplicates. However, concerted evolution does not necessarily homogenize a gene cluster. Gene conversion can both homogenize and diversify among paralogues, as was shown for the gene family *hsp70* in *Drosophila*[80].

Intuitively one might think that gene conversion and recombination should tend to break up linkage disequilibrium. But this does not seem to be this simple. The human olfactory receptor (OR) gene cluster is another gene family suggested to evolve under concerted evolution with gene conversion events [81]. Linkage disequilibrium is highly significant in the centromeric part of the gene cluster, whereas no linkage disequilibrium could be observed in the telomeric part of the cluster. These considerable differences suggest the presence of several recombination hotspots within the OR cluster.

In terms of number of different alleles, MHC diversity was similar at the two BLB loci, however, BLB2 revealed higher heterozygosity and was less variable in terms of nucleotide diversity than BLB1. Signals of positive selection were found on both the BLB1 and BLB2 locus. Elevated *dN/dS* ratios were obtained at the PBR of both loci, though only significant for the longer BLB2 fragment. Maximum likelihood analyses confirmed that models allowing for positive selection fitted our data significantly better than neutral models. With these models, four identical codons were identified to be under significant positive selection in BLB1 and BLB2, and two additional positions were identified for the BLB2 locus. All of these positively selected codon positions were either congruent or directly adjacent to a peptide binding site as identified by Tong et al. [73] for human MHC class II molecules. This congruence emphasises the structural similarity of codon positions involved in peptide binding throughout evolutionary lineages. However, it has been suggested the methods used here might be prone to overestimate positive selection, particularly when recombination rate is high [82].

A different pattern of selection was found, however, regarding neutrality tests as Tajima’s D. We found a negative Tajima’s D value for BLB1, indicative of purifying selection, and a positive Tajima’s D for BLB2 indicating positive or balancing selection, although not significant in both cases. In a sliding window analysis of Tajima’s D comparing BLB1 and BLB2 it became more apparent that these different selection footprints are due to differences at some specific nucleotide sites only, while at other positions positive selection between the two loci coincides. These opposite but vague signals of differential selection at BLB1 and BLB2 made us explore this further. Worley et al. [22] found likewise a negative Tajima’s D for BLB1 and positive value for BLB2 (both n.s.) in a captive population of red jungle fowl but the authors points out that a negative Tajima’s D could also be a result of a population bottleneck. As a further comparison, we obtained domestic chicken BLB1 and BLB2 sequences from GenBank and calculated Tajima’s D the same way as we did for the black grouse sequences. This analysis repeated the observation of positive Tajima’s D for BLB2 and negative for BLB1 (data not shown). We also observed that theta *k* and π were higher in BLB1 than BLB2 in the domestic chicken, which was likewise observed for black grouse. In conclusion, we interpret this as a repeated pattern of differential selection on BLB1 and BLB2 in galliform birds.

Detecting recombination or gene conversion events using statistical methods can be highly problematic, in particular when small fragments are transferred or the gene conversion rate is too high. Hence, we have to keep in mind that a lack of evidence is no evidence for absence. To minimize the risk of missing the footprint of gene conversion, it is recommended to use multiple statistical methods for detection [58]. Following this recommendation we applied a set of seven statistical methods, which have been evaluated to be powerful and accurate for different conditions and scenarios [59]. While two of the methods (RDP and GENECONV) failed to detect most recombination signals, the other methods detected recombination or gene conversion both within and 'between the two BLB loci. As an additional hint for the occurrence of gene conversion, we detected significantly higher *d*_*N*_ and *d*_*S*_ values at the PBR sites compared to the non-PBR sites at both loci. This situation is likely to be created by a combination of positive selection and gene conversion [78,83]. Gene conversion events transferring advantageous non-synonymous substitutions at the PBR are positively selected, and in doing so synonymous substitutions are carried along. This way, synonymous substitutions appear more often at the PBR than expected by point mutations under neutrality. We conclude that both intra- and interlocus genetic exchange play an important role in shaping the black grouse MHC class II.

The 3′UTRs between BLB1 versus BLB2 differ in domestic chicken as well as between the corresponding DAB1 and DAB2 loci in pheasants in both length and nucleotide composition, so that the sequences cluster together as orthologous genes rather than according to species [47]. We have observed similar differences in the 3′UTRs between black grouse BLB1 and BLB2 and could prove orthology of the BLB1 gene between the black grouse, chicken and pheasant, and orthology of the BLB2 gene between the three species. The origin of the pheasant like birds Phasianoidea is estimated to approximately 40 million years ago [84] and chicken and black grouse/turkey diverged approximately 30 million years ago [85]. It seems that the duplication of BLB1 and BLB2 is a case of pre-speciation duplication that has arisen in the ancestral species before the split into chicken, pheasant and black grouse. The observed differences in the length of the 3′UTRs between the two loci may also reflect a difference in function [86].

In all other phylogenetic reconstructions based on the exon 2, the third codon positions of exon 2 and the exon 3, sequences clustered species-specific and not locus-specific. This is indication for frequent interlocus genetic exchange homogenizing sequences between the paralogous loci, as suggested by Wittzell et al. [47]. The fact that the whole exon 2 sequences and third codon positions of the exon 2 networks present similar phylogenetic relations contains more indication for an early duplication in the evolutionary history of BLB1 and BLB2. Under convergent selection on the two loci, the two phylogenies would reveal deviating patterns [34]. A phylogenetic tree based on the codon sequences should reflect functional similarities, whereas the third codon positions are expected to mirror the neutral gene history. Under the early duplication hypothesis, in contrast, a phylogeny based on the third codon positions will match the phylogenetic relation based on the whole codon sequences, as in our case. Note that in this analysis all third codon positions are considered and not only the synonymous positions at the PBR, which are possibly under a selective sweep of the positions under selection, as discussed earlier.

In summary, we infer that the BLB gene duplicated before the species divergence into chicken, black grouse and pheasant and thus is a case of pre speciation duplication. Further, we conclude that BLB1 and BLB2 in black grouse are subjected to homogenizing concerted evolution due to inter-genetic exchange between loci after species divergence. Different selection patterns indicated for BLB1 and BLB2 may be a sign of different immunogenetic functions. Both BLB1 and BLB2 have been under balancing selection during their evolutionary history. Nevertheless, it is likewise possible that, at present, balancing selection may be operating directly only on BLB2. Because of the tight linkage between the loci, BLB1 would be hitchhiking with BLB2.

## Conclusions

We have presented a powerful single locus genotyping method for amplifying MHC class IIB loci in black grouse. This method will allow exploring correlations between MHC heterozygosity and reproductive success, mate choice and disease resistance in this and related species.

It is evident that both BLB1 and BLB2 are expressed and under balancing selection in black grouse. Even though we found some differences between the loci in selection patterns, the similarities between loci were apparent. In fact, our data show inter-locus genetic exchange between BLB1 and BLB2. The loci are in linkage disequilibrium, which is in line with what have been stated regarding tightly coevolving genes within MHC under the minimal essential MHC hypothesis [31,36]. Our results support the conclusion that MHC form and function in birds derived from studies on the domesticated chicken are not artefacts of the domestication process. However, the data from black grouse do suggest more recombination than previously observed in chicken (but see [39]). These data are important for understanding how the MHC of birds and other non-mammalian vertebrates have evolved.

The duplicated genes BLB1 and BLB2 are a case of early duplication and have co-evolved in a concerted way by interlocus gene exchange not only in chicken but also in black grouse. This supports the so far non-tested hypothesis of concerted evolution of BLB1 and BLB2 in galliformes [47].

## Competing interests

The authors declare that they have no competing interests.

## Authors’ contributions

TS and JH conceived and designed the study, TS and BW carried out the molecular genetic studies. TS, YML and BW performed the statistical analyses and interpretation of the data. The manuscript was written by TS and YML, with input from JH and BW. All authors read and approved the final manuscript.

## Supplementary Material

Additional file 1 **MHC primers and corresponding amplification lengths.** The fragment column highlights the locus and region amplified with each primer pair. The PCR product length is given without primers. Click here for file

Additional file 2 **Best evolutionary models estimated by Aikaike’s information criterion in jModelTest.** The models were used to construct phylogenetic trees. Click here for file

Additional file 3 **Alignment of black grouse MHC class II B exon 2 nucleotide sequences.** Locus-designated sequences derived in the present study are indicated with 125 bp and 251 bp. Sequences indicated as BLB* are cloned in previous studies and not designated to locus, for example 202 bp cDNA sequences (from individual D870, see Table 1). Sequences likely to be from the same allele are grouped by grey shading. Click here for file

Additional file 4 **Supplement for the test for positive selection.** Likelihood values and parameter estimates for the different models calculated with CODEML implemented in PAML 4.6 [74]. M = model, lnL = Log-likelihood value. In the neutral model M0, ω is equivalent to averaged *dN/dS*. Dark grey shade highlights the significantly best models. Click here for file

Additional file 5 **Neighbour Joining tree for exon 3.** BLB1 and BLB2 sequences derived from black grouse (fosmid individual JHGO 213 [48] [GenBank JQ028669] and chicken [GenBank AB268588]. Goose [GenBank EU999169] was used as an outgroup. Click here for file
