# Supplementary material for: Evolutionary history of black grouse major histocompatibility complex class IIB genes revealed through single locus sequence-based genotyping
Source: BMC Genet. 2013 Apr 24;14:29. doi: 10.1186/1471-2156-14-29 (PMC3652749; doi:10.1186/1471-2156-14-29)
Supplement: Additional file 1 — MHC primers and corresponding amplification lengths. The fragment column highlights the locus and region amplified with each primer pair. The PCR product length is given without primers. [file 1471-2156-14-29-S1.docx]

Additional file 1. MHC primers and corresponding sequences lengths. The fragment column highlights the locus and region amplified with each primer pair. The PCR product length is given without primers.

| **Fragment** | **Primer** | **Sequence 5'-3'** | **Product length (bp)** | |
| --- | --- | --- | --- | --- |
| BLB1 | C275* | GGTTCCAGGTGCAAGGCGATGGTCTCTGTGCATACCT | | 1870 |
|  | NBG262** | GCACTTCGACAGCGAAGTGGGGAAA | |  |
| BLB2 | preBLB2F | GGAGGCATCTGGATGACAGT | | 1319 |
|  | BLBex3R | GTAGAAGCCCGTCACGTAGC | |  |
| BLB_125_ | RNA F 1a | GACAGCGAAGTGGGGAAATA | | 125 |
|  | RNA R 1a | CGCTCCTCTGCACCGTGA | |  |
| BLB_251_ | Vorinex2 | TGCCCTCTGCCCGCAGCGTTCTT | | 251 |
|  | Postex2 | GCACTCACCGCTCCTCTGCA | |  |

* [[42](#_ENREF_42)]

** A modification of primer C262 in [[42](#_ENREF_42)].
