# Supplementary material for: Evolutionary history of black grouse major histocompatibility complex class IIB genes revealed through single locus sequence-based genotyping
Source: BMC Genet. 2013 Apr 24;14:29. doi: 10.1186/1471-2156-14-29 (PMC3652749; doi:10.1186/1471-2156-14-29)
Supplement: Additional file 2 — Best evolutionary models estimated by Aikaike’s information criterion in jModelTest. The models were used to construct phylogenetic trees. [file 1471-2156-14-29-S2.docx]

Additional file 2. Best evolutionary models estimated by Aikaike’s information criterion (AIC) in jModelTest. The models were used to construct phylogenetic trees.

| **Analysis** | **AIC** | **Evolutionary model** | **Gamma value** |
| --- | --- | --- | --- |
| Exon 2 | 4351.8676 | TrN+I+G | 2.3730 |
| Exon 2, codon 3 | 710.7717 | TrN+G | 1.1600 |
| Exon 3 | 588.5412 | TrN | - |
| UTR | 453.8094 | TrN+I | - |
