# Supplementary material for: Evolutionary history of black grouse major histocompatibility complex class IIB genes revealed through single locus sequence-based genotyping
Source: BMC Genet. 2013 Apr 24;14:29. doi: 10.1186/1471-2156-14-29 (PMC3652749; doi:10.1186/1471-2156-14-29)
Supplement: Additional file 3 — Alignment of black grouse MHC class II B exon 2 nucleotide sequences. Locus-designated sequences derived in the present study are indicated with 125 bp and 251 bp. Sequences indicated as BLB* are cloned in previous studies and not designated to locus, for example 202 bp cDNA sequences (from individual D870, see Table 1). Sequences likely to be from the same allele are grouped by grey shading. [file 1471-2156-14-29-S3.docx]

**Additional file 3**: **Alignment of black grouse MHC class II B exon 2 nucleotide sequences**

Locus-designated sequences derived in the present study are indicated with 125 bp and 251 bp. Sequences indicated as BLB* are cloned in previous studies and not designated to locus, for example 202 bp cDNA sequences (from individual D870, see Table 1). Sequences likely to be from the same allele are separated by grey shade.

10 20 30 40 50 60 70 80 90 100 110 120 130

....|....|....|....|....|....|....|....|....|....|....|....|....|....|....|....|....|....|....|....|....|....|....|....|....|....|

**BLB2_251-01A**  **--CCTCGACGGTATGATAGTTGAGTGCCAATTCCTGAACGGCACCGAGCAGGTGAGGCTTCTGGAGAGGCAGATCTACAACCGGCAGCAGTTCGCGCACTTCGACAGCGAAGTGGGGAAATATGTGGCCG**

**BLB*_01_cDNA_EF174544** **---------------------------------------------------...............................................................................**

**BLB2_125-01**  **--------------------------------------------------------------------------------------------------------------------------........**

**BLB2_251-01B**  **--.........................................................................................A......................................**

**BLB2_251-01C**  **--....T...........................................................................................................................**

**BLB2_251-02**  **--................................................................................................................................**

**BLB*_02_cDNA_EF174545** **---------------------------------------------------...............................................................................**

**BLB2_125-02**  **--------------------------------------------------------------------------------------------------------------------------........**

**BLB*_03_cDNA_EF174546** **---------------------------------------------------......T................T.......................................................**

**BLB1_125-03**  **--------------------------------------------------------------------------------------------------------------------------........**

**BLB2_251-04B**  **--...T..T...G.....T..........C.A.................G........A.G........G.C...C...............A......................................**

**BLB2_125-04**  **--------------------------------------------------------------------------------------------------------------------------........**

**BLB*_04_cDNA_EF174547** **---------------------------------------------------.......A.G........G.C.....................AT...................................**

**BLB1_125-04**  **--------------------------------------------------------------------------------------------------------------------------........**

**BLB1_125-05**  **--------------------------------------------------------------------------------------------------------------------------........**

**BLB1_125-06**  **--------------------------------------------------------------------------------------------------------------------------........**

**BLB2_251-07**  **--...T..A...G.....T..........C.A.................G........A.G........G.C...C...............A......................................**

**BLB2_125-07**  **--------------------------------------------------------------------------------------------------------------------------........**

**BLB2_251-09**  **--...T..A...G.....T..........C.A.................G........A.G........G.C...C...............A......................................**

**BLB*_09_EF174552.1**  **--------------------------------------------------------------------------------------------------------------------------........**

**BLB2_125-09**  **--------------------------------------------------------------------------------------------------------------------------........**

**BLB2_251-10**  **--....T.....G......C.........C.A................................TC...A.CG....................AT...................................**

**BLB2_125-10**  **--------------------------------------------------------------------------------------------------------------------------........**

**BLB1_125-11**  **--------------------------------------------------------------------------------------------------------------------------........**

**BLB2_251-12**  **--...T..A...G.....T..........C.A.................G........A.G........G.C...C...............A......................................**

**BLB2_125-12**  **--------------------------------------------------------------------------------------------------------------------------........**

**BLB1_125-14**  **--------------------------------------------------------------------------------------------------------------------------........**

140 150 160 170 180 190 200 210 220 230 240 250

....|....|....|....|....|....|....|....|....|....|....|....|....|....|....|....|....|....|....|....|....|....|....|....|...

**BLB2_251-01A**  **ATACAGCGCTGGGAGAGCTGCAAGCTGAATACTGGAACAACAACACTGAGCGTCTGGAGTATGCACGGGGTGCAGTGGATACGTACTGCCGGCACAACTACGGGGTGTTTGAGCCCTTCACGG**

**BLB*_01_cDNA_EF174544** **...........................................................................................................................**

**BLB2_125-01**  **.....................................................................................................................------**

**BLB2_251-01B**  **...........................................................................................................................**

**BLB2_251-01C**  **...........................................................................................................................**

**BLB2_251-02**  **....................................................................................T......................................**

**BLB*_02_cDNA_EF174545** **....................................................................................T......................................**

**BLB2_125-02**  **....................................................................................T................................------**

**BLB*_03_cDNA_EF174546** **.....C..................................................................AG.....C.GA.T.................~..GT.GG.GAG..C.TCAC.**

**BLB1_125-03**  **.....C..................................................................AG.....C.GA.T.....................TGGG.......------**

**BLB2_251-04B**  **.....C...........................................ATA.........C.T.......T.......C....T...................A.TC.G.............**

**BLB2_125-04**  **.....C...........................................ATA.........C.T.......T.......C....T...................A.TC.G.......------**

**BLB*_04_cDNA_EF174547** **.....C...........................................ATA.........C.T.......T.......C....T...................A.TC.G.............**

**BLB1_125-04**  **.....C...........................................ATA.........C.T.......T.......C....T...................A.TC.G.......------**

**BLB1_125-05**  **................................AT................TA......AATAAA.A..AA..AG.....C....T................................------**

**BLB1_125-06**  **.....C..................................TG....CC..TT.A.T.....CAA..A....CAG.....C.GA.......................TG.G.......------**

**BLB2_251-07**  **.....C..........................AT.......G........TA..........AG........AG.....C.GA.....................A.TC.G.............**

**BLB2_125-07**  **.....C..........................AT.......G........TA..........AG........AG.....C.GA.....................A.TC.G.......------**

**BLB2_251-09**  **.....C...............C...................G........TA..........AG........AG.....C.GA.......................TGGG.............**

**BLB*_09_EF174552.1**  **.....C...............C...................G........TA..........AG........AG.....C.GA.......................TGGG.......------**

**BLB2_125-09**  **.....C...............C...................G........TA..........AG........AG.....C.GA.......................TGGG.......------**

**BLB2_251-10**  **.....C..........................A.................TA.........CTT.......T.......C....T......................AA..............**

**BLB2_125-10**  **.....C..........................A.................TA.........CTT.......T.......C....T......................AA........------**

**BLB1_125-11**  **.....C...........................................ATA.........C.T.......T.......C....T......................AA........------**

**BLB2_251-12**  **.....C...............C...................G........TA..........AG........AG.....C.GA.......................TG.G.............**

**BLB2_125-12**  **.....C...............C...................G........TA..........AG........AG.....C.GA.......................TG.G.......------**

**BLB1_125-14**  **.....C...............C.......................................CAA.......T.......C.GA.T................................------**
