# Supplementary material for: Evolutionary history of black grouse major histocompatibility complex class IIB genes revealed through single locus sequence-based genotyping
Source: BMC Genet. 2013 Apr 24;14:29. doi: 10.1186/1471-2156-14-29 (PMC3652749; doi:10.1186/1471-2156-14-29)
Supplement: Additional file 4 — Supplement for the test for positive selection. Likelihood values and parameter estimates for the different models calculated with CODEML implemented in PAML 4.6 [74]. M = model, lnL = Log-likelihood value. In the neutral model M0, ω is equivalent to averaged dN/dS. Dark grey shade highlights the significantly best models. [file 1471-2156-14-29-S4.docx]

Additional file 4. Test for positive selection: likelihood values and parameter estimates for the different models calculated with CODEML implemented in PAML 4.6 [[71](#_ENREF_71)]. M=model, lnL=Log-likelihood value. In the neutral model M0, ω is equivalent to averaged *dN/dS*. Dark grey shade highlights the significantly best models.

| **Locus** | **M** | **lnL** | **Parameter estimate** |
| --- | --- | --- | --- |
| **BLB1_125_** | M0 | -406.28 | ω=0.67 |
|  | M1a | -391.72 | p_0_ =0.68, p_1_= 0.32, ω_0_=0.06, ω_1_=1 |
|  | M2a | -374.48 | p_0_ =0.47, p_1_= 0.43, p_2_= 0.10, ω_0_=0.01, ω_1_=1, ω_2_=26.91 |
|  | M7 | -392.26 | p=0.02, q=0.02 |
|  | M8 | -374.48 | p_0_ =0.90, (p_1_= 0.10), p= 0.01, ω=27,49, q=0.01 |
|  |  |  |  |
| **BLB2_125_** | M0 | -341.91 | ω=0.90 |
|  | M1a | -328.82 | p_0_ =0.67, p_1_= 0.33, ω_0_=0, ω_1_=1 |
|  | M2a | -317.37 | p_0_ =0.58, p_1_= 0.28, p_2_= 0.14, ω_0_=0, ω_1_=1, ω_2_=16.45 |
|  | M7 | -328.89 | p=0.01, q=0.01 |
|  | M8 | -317.38 | p_0_ =0.86, (p_1_= 0.14), p= 0.01, ω=15,87, q=0.01 |
|  |  |  |  |
| **BLB2_251_** | M0 | -636.73 | ω=1.09 |
|  | M1a | -625.20 | p_0_ =0.56, p_1_= 0.44, ω_0_=0, ω_1_=1 |
|  | M2a | -611.66 | p_0_ =0.43, p_1_= 0.48, p_2_= 0.09, ω_0_=0, ω_1_=1, ω_2_=15.52 |
|  | M7 | -625.31 | p=0.01, q=0.01 |
|  | M8 | -611.75 | p_0_ =0.91, (p_1_= 0.09), p= 0.01, ω=17,05, q=0.01 |
|  |  |  |  |
| **BLB1&2_125_** | M0 | -522.32 | ω=0.86 |
|  | M1a | -492.89 | p_0_ =0.69, p_1_= 0.31, ω_0_=0.04, ω_1_=1 |
|  | M2a | -470.19 | p_0_ =0.55, p_1_= 0.32, p_2_= 0.12, ω_0_=0.03, ω_1_=1, ω_2_=11.94 |
|  | M7 | -494.41 | p=0.07, q=0.13 |
|  | M8 | -470.22 | p_0_ =0.88, (p_1_= 0.12), p= 0.01, ω=12.34, q=0.02 |
