# Supplementary material for: Evolutionary history of black grouse major histocompatibility complex class IIB genes revealed through single locus sequence-based genotyping
Source: BMC Genet. 2013 Apr 24;14:29. doi: 10.1186/1471-2156-14-29 (PMC3652749; doi:10.1186/1471-2156-14-29)
Supplement: Additional file 5 — Neighbour Joining tree for exon 3. BLB1 and BLB2 sequences derived from black grouse (fosmid individual JHGO 213 [48] [GenBank JQ028669] and chicken [GenBank AB268588]. Goose [GenBank EU999169] was used as an outgroup. [file 1471-2156-14-29-S5.docx]

Additional file 5. Neighbour Joining tree for exon 3 BLB1 and BLB2 loci with sequences derived from black grouse (fosmid individual JHGO 213, Wang et al. in review) [GenBank JQ028669].and chicken [GenBank AB268588]. Goose [GenBank EU999169] was used as an outgroup.

**
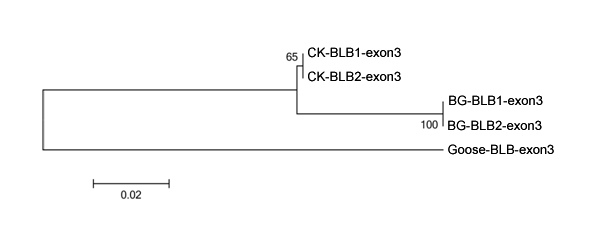
**
